# Influence of Fusion Temperature on Nonisothermal Crystallization Kinetics of Polyamide 6

**DOI:** 10.3390/polym15081952

**Published:** 2023-04-20

**Authors:** Ahmed Nasr, Petr Svoboda

**Affiliations:** Department of Polymer Engineering, Faculty of Technology, Tomas Bata, University in Zlin, Vavreckova 5669, 76001 Zlin, Czech Republic; svoboda@utb.cz

**Keywords:** polyamide 6, DSC, optical microscope, nonisothermal crystallization, fusion temperature, cooling function

## Abstract

The effect of fusion temperature and duration on the nonisothermal crystallization kinetics of polyamide 6 (PA6) was investigated using differential scanning calorimetry (DSC) and a polarized optical microscope (OM). The rapid cooling method involved heating the polymer above its melting point, holding it at this temperature to ensure complete melting, and then rapidly cooling it to the crystallization temperature. By monitoring the heat flow during cooling, the crystallization kinetics of PA6 were characterized, including the degree of crystallinity, crystallization temperature, and crystallization rate. The study found that changing the fusion temperature and duration significantly impacted the crystallization kinetics of PA6. Increasing the fusion temperature decreased the degree of crystallinity, with smaller nucleation centers requiring a higher degree of supercooling for crystallization. The crystallization temperature shifted towards lower temperatures, and the crystallization kinetics slowed down. The study also found that lengthening the fusion time raised the relative crystallinity, but any further increase did not result in a significant change. The study showed that an increase in fusion temperature led to a longer time needed to reach a given level of crystallinity, reducing the crystallization rate. This can be explained by the thermodynamics of the crystallization process, where higher temperatures promote molecular mobility and crystal growth. Moreover, the study revealed that decreasing a polymer’s fusion temperature can lead to a greater degree of nucleation and faster growth of the crystalline phase, which can significantly impact the values of the Avrami parameters used to characterize the crystallization kinetics.

## 1. Introduction

Polyamide 6 (PA6) is a semi-crystalline thermoplastic material with high strength, toughness, and stiffness, making it an attractive material for various applications [1,2]. During the processing of PA6, the fusion temperature plays a crucial role in controlling the crystallization behavior of the polymer [3,4]. The crystallization behavior of polyamide 6 is influenced by various factors such as temperature, cooling rate, and molecular weight [5]. Most polyamides (nylons) are characterized by a linear structure with repeating amide units. These amide groups enable hydrogen bonding within the polymer chain, which plays a critical role in the physical and chemical properties of the material [2,6]. The formation of hydrogen bonds within PA6 results in a strong, rigid, and crystalline structure that contributes to the material’s high strength, stiffness, and thermal stability. This unique arrangement of chemical bonds also imparts excellent chemical resistance and low moisture absorption to PAs, making them ideal for various applications in industries such as textiles, automotive, and electronics.

Polymer crystallization involves the partial alignment of molecular chains, forming ordered regions called lamellae [7]. These folded chains combine to form larger spheroidal structures known as spherulites. This is a significant phenomenon in polymers as it influences the physical and mechanical properties of the material, such as its stiffness, strength, and resistance to deformation [8]. The way in which a polymer crystallizes, including its crystallinity and crystallization kinetics, is extremely important when designing and optimizing the technological process. This is because the crystallization behavior of a polymer has a significant impact on the end-use properties of products that are created through injection molding [9]. The crystallization process in polymers begins when the polymer is cooled after melting, stretched mechanically, or evaporated using solvents. The crystallization process impacts several properties of the polymer, including its optical, mechanical, thermal, and chemical properties [10]. The degree of crystallinity, which various analytical methods can determine, usually falls between 10% and 80%, and polymers with such a range are referred to as “semi-crystalline”. In semi-crystalline polymers, the degree of crystallinity is not the only factor determining the material’s properties, as the size and orientation of the molecular chains also play a significant role [11].

The effect of fusion temperature on the nonisothermal crystallization of semi-crystalline polymers has been studied extensively, with several research papers reporting on its impact on the degree of crystallinity and the crystalline structure of the material during nonisothermal crystallization [3,4,12,13]. Ziabicki and Alfonso [12] explain the kinetics of polymer crystallization by breaking down the process into three stages: nucleation, growth, and termination. According to this theory, the crystal growth rate is directly proportional to the diffusion rate of polymer chains into the crystal lattice. Alfonso and Ziabicki [3] researched how the fusion temperature and time affect polypropylene’s isothermal crystallization kinetics. The authors found that increasing the fusion temperature leads to slower overall crystallization but a higher final degree of crystallinity due to changes in the morphology and molecular ordering of the polymer crystals.

On the other hand, increasing the duration of fusion leads to faster nucleation and crystal growth. The researchers attributed these effects to changes in the polypropylene’s molecular structure due to the melting and cooling process. Specifically, they suggested that the high temperatures used during fusion caused some of the crystalline regions of the material to break down, leading to a decrease in the overall degree of crystallinity. Meanwhile, longer fusion times allowed for more time for the remaining crystalline regions to reform and grow, increasing the degree of crystallinity. To our best knowledge, this study has not been conducted for polyamide 6. Overall, these studies indicate that the fusion temperature of semi-crystalline polymers can significantly impact the nonisothermal crystallization behavior of the material. The degree of crystallinity, crystalline structure, and spherulite morphology of the material can be influenced by the fusion temperature, cooling rate, and other factors. Understanding these effects can aid in developing new materials with desired properties.

A new phenomenon has been reported, which involves a memory effect in the crystallization rate of PA6 from the molten state that is influenced by its processing history [14]. Differential scanning calorimetry (DSC) and optical microscopy are the main techniques used to show that processing variables can control the melt crystallization behavior of PA6. As the process of crystallization progresses, the surface area increases, and the kinetics of growth can become more favorable. If a constant cooling rate is applied to the crystallization process, there can be initial supersaturation buildup where no surface area is available for growth. This buildup can result in fast and unpredictable crystallization kinetics, with nucleation often becoming the dominant factor.

Some authors have investigated the crystallization behavior of PA6 [5,15,16,17]. However, less attention has been paid to studying the influence of fusion temperature on the crystallization kinetics of PA6 since it affects not only the morphology of semi-crystalline polymers and the crystalline structure but also the final physical properties end-use properties of the polymers. This article examined the impact of fusion temperature on the nonisothermal crystallization kinetics of PA6 using differential scanning calorimetry (DSC). The researchers used various models, including Avrami, Nakamura, and Ozawa models, to analyze the crystallization kinetics of PA6. The goal was to better understand the factors influencing the crystallization behavior of PA6, which is a crucial aspect in designing and processing this polymer material [18,19,20]. The results showed that the fusion temperature significantly impacts the crystallization rate and the degree of crystallinity of PA6.

Overall, this work aimed to study the effect of the fusion temperature on the nonisothermal crystallization process of PA6 by understanding the overall crystallization kinetics, growth rate, and spherulitic morphology in a wide range of crystallization temperatures by applying different cooling rates.

## 2. Experimental Section

**Figure 1:**
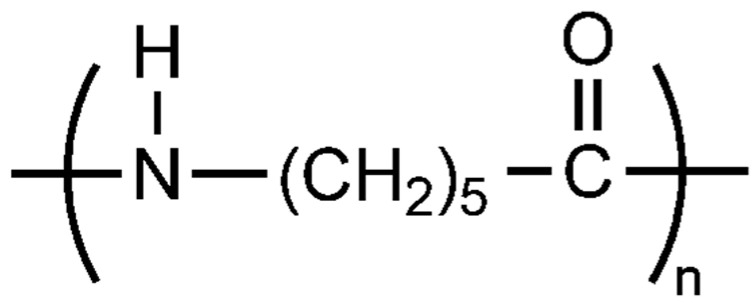
Molecular structure of PA6.

Polyamide 6 was supplied by DSM Company (Genk, Belgium) with a trading name Akulon^®^F232-D, PA6. Figure 1 shows the molecular structure of PA6, and the main characteristics of the material are listed in Table 1. PA6 granules were used directly for the measurement itself. However, drying the granules due to moisture was necessary and took place at 80 °C for 12 h. The samples were then prepared as a thin film, melting the PA6 granules at 200 °C, and then pressed between two slides. After cooling, a thin film with a thickness of about 120 μm was formed. Different specimens were used in each experiment.

A polarizing optical microscope determined nonisothermal crystallization (BHA-P Olympus, Olympus Global, Tokyo, Japan) attached to a temperature controller (Line LK 600-PM, Linkam Scientific Instruments, London, UK). The PA6 samples were melted at 200 °C for 2 min. They were then cooled at different cooling rates (15, 20, and 25 °C/min) and spherulites grew.

The behavior of the PA6 heat flow was determined using differential scanning calorimetry (DSC) on Mettler Toledo’s DSC instrument, Greifensee, Switzerland. About 20 mg of the prepared samples were prepared for the measurement which was carried out with nitrogen access (30 mL/min) to avoid significant thermal degradation. The samples were melted at various fusion temperatures from 225 °C to 245 °C for 2 min and then cooled to 60 °C. The effect of cooling rates (15, 20 and 25 °C/min) was also monitored.

To prevent PA6 degradation, we utilized a maximum temperature of 245 °C throughout our experimentation, as degradation of PA6 typically begins at temperatures exceeding 300 °C [21].

## 3. Theoretical Background

### 3.1. Avrami Analysis

The Avrami model is a mathematical model commonly used to study polymers’ isothermal crystallization kinetics. The model proposes that the crystallization rate in a polymer system is proportional to the amount of amorphous material remaining at any given time. The model equation includes the fraction of crystalline material formed at a given time, the isothermal crystallization temperature, a rate constant that depends on the polymer and crystallization conditions, and an Avrami exponent that describes the mechanism of crystallization. The equation representing the model is as follows, Equation (1) [18]:

(1) $$1-X_{t}=exp\left(-kt^{n}\right)$$

where *n* is the Avrami exponent, and *k* is the Avrami rate constant. Both *n* and *k* depend on the rate of growth mechanisms and nucleation of the spherulites.

The nonisothermal crystallization parameters obtained from the DSC are used to calculate the crystallinity $X_{t}$ from the area of the exothermic peak within the crystallization time t, then divided by the total area under the peak:

(2) $$X_{t}=\frac{\int_{0}^{t}\left(\frac{dH}{dt}\right)dt}{\int_{0}^{\infty }\left(\frac{dH}{dt}\right)dt}\;\;\;\;\;$$

where the numerator represents the heat generated at time *t* and the denominator means the total heat generated up to complete crystallization.

Avrami constants can be evaluated by the linear regression, as described in Equation (1), then applying double logarithmic form as follows:

(3) $$ln\left[-ln\left(1-X_{t}\right)\right]=lnk+n\;lnt\;\;\;\;\;\;\;\;\;\;\;\;\;$$

The *n* and *k* values are obtained using Equation (3) from the slope and intercept of the linear regression line. Several authors have employed the Avrami equation to assess the rate of polymer crystallization in nonisothermal conditions.

### 3.2. Nakamura Model

The Nakamura model, introduced in 1973 [20], is a well-established model for characterizing the nonisothermal crystallization of polymers. This model considers both temperature and the extent of crystallization in the polymer and the kinetics of crystallization described by the Avrami equation. The crystallization rate is determined by the temperature-dependent rate constant and the Avrami exponent, which characterizes the three-dimensional growth of the crystal. The rate of crystallization can be expressed using the following equation, Equation (4):

(4) $$X_{t}={\;}_{}1-\exp\left[-{\left(\int_{0}^{t}K\left(T\right)dt\right)}^{n}\right]$$

where *K(T)* is related to *k(T)* in Equation (1) and can be calculated by the following equation, Equation (5) [22]:

(5) $$K\left(T\right)=k{\left(T\right)}^{1/n}$$

Additionally, the Nakamura model accounts for the effect of temperature on the crystallization process through the nonisothermal term, which reflects the sensitivity of the crystallization rate to changes in temperature.

### 3.3. Ozawa Model

The Ozawa model is an extension of the Avrami model, which accounts for the effects of crystallite size distribution on the kinetics of nonisothermal crystallization of polymers [19]. The equation is given by:

(6) $$X=1-e^{{\left(\frac{K}{\Phi^{m}}\right)}^{}}$$

where *X* represents crystallinity, *K* is Ozawa’s rate constant of crystallization, *m* is the Ozawa parameter representing the growth and nucleation of crystals, and *Φ* denotes the cooling rate. After two logarithms of Equation (1), Ozawa’s equation takes the following form:

(7) $$\log\left[-ln\left(1-X\right)\right]=logK-mlog\Phi$$

## 4. Results and Discussion

The nonisothermal crystallization kinetics of polyamide 6 (PA6) was investigated through a rapid cooling method using differential scanning calorimetry (DSC). This technique involves heating the polymer above its melting point, holding it at this temperature to ensure complete melting, and then rapidly cooling it to the crystallization temperature. By monitoring the heat flow during cooling using DSC, the crystallization kinetics of PA6 can be characterized, including the degree of crystallinity, crystallization temperature, and crystallization rate. This approach is commonly used in materials science research to study the thermal behavior of polymers and has been proven effective in characterizing the crystallization kinetics of various types of polymers, including PA6.

**Figure 2:**
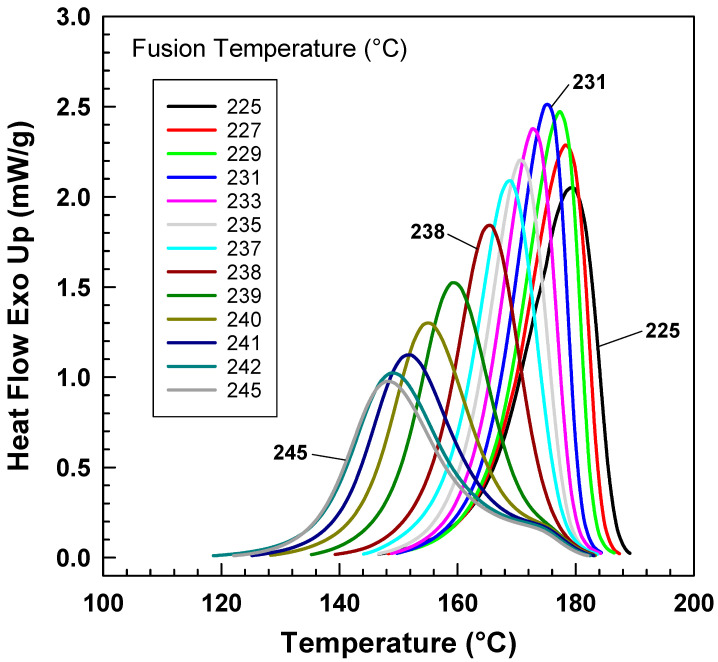
Exothermic heat flow of PA6 from DSC after various fusion temperatures at a cooling rate of 25 °C/min.

Figure 2 illustrates the impact of different fusion temperatures on the heat flow [23], in which PA6 samples were heated at various fusion temperatures from 225 °C to 245 °C for 2 min and then were cooled at a cooling rate of 25 °C/min. The graph demonstrates that with increasing fusion temperature, the exothermic trace becomes narrower and shifts toward lower temperatures, then it becomes wider but still moves toward lower temperatures. At lower fusion temperatures, some crystals remain unmelted, which act as nucleation centers. As the fusion temperature increases, more crystals melt, leading to smaller nucleation centers. Consequently, crystallization becomes more challenging, requiring a higher degree of supercooling. Therefore, the crystallization temperature shifts towards lower temperatures. This change is most noticeable in the temperature range of 225–242 °C, and then the peak remains at approximately the same temperature as the fusion temperature increases.

The presence of shoulder on some of the heat flow curves suggests the presence of “transcrystallinity” described by Freire et al. [24] meaning differences in crystallization kinetics on the surface (in contact with aluminum pan) and inside the pellet. Thickness of the sample seems to be very important. For our polarized optical microscopy measurement, it was necessary to use thicker sample (more than 100 μm) in order to be able to observe spherulites.

However, in terms of crystallization kinetics, the inverse value of crystallization is preferable. Conversely, this value decreases with increasing fusion temperature, indicating that crystallization kinetics are slowing.

**Figure 3:**
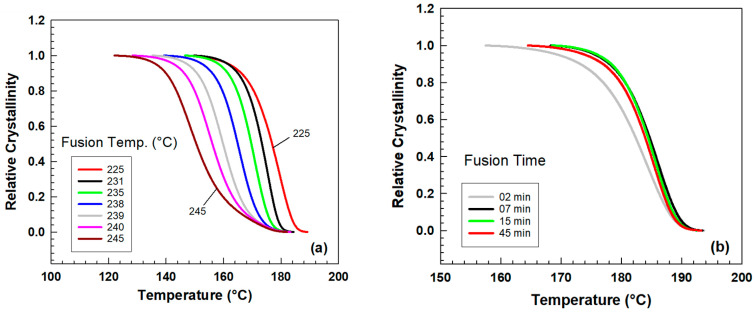
Relative degree of crystallinity as a function of temperature for PA6 (**a**) fusion temperature from 225 °C to 245 °C at 25 °C/min (**b**) fusion time 2, 7, 15, and 45 min at constant T_f_ = 239 °C.

The degree of crystallinity is affected by changing the fusion temperatures and time of fusion [16,25,26,27]. Relative crystallinity can be calculated by the following equation (Equation (2)), [28]. Figure 3 illustrates how the nonisothermal crystallization kinetics of PA6 is influenced by varying the fusion temperature and duration. When the fusion temperature is increased, the temperature at which relative crystallinity is achieved decreases (Figure 3a). Meanwhile, lengthening the fusion time from 2 to 7 min raises the relative crystallinity and causes the curve to shift toward higher temperatures (Figure 3b). However, any further increase in the fusion time does not result in a significant change in the relative crystallinity. It seems that 2 min fusion time is not sufficient to erase previous processing history. In the literature [29,30], 5 min fusion time is usually used prior to crystallization.

Equation (2) can be modified into crystallization time t and t_0_ instead of the crystallization temperature *T* and $T_{0}$*,* respectively. The relationship between the crystallization temperature and the crystallization time can be summarized in the following equation (Equation (8)) [31].

(8) $$t=\frac{T_{0}-T}{C}$$

where *C* is the cooling rate applied to the nonisothermal crystallization kinetics of PA6.

**Figure 4:**
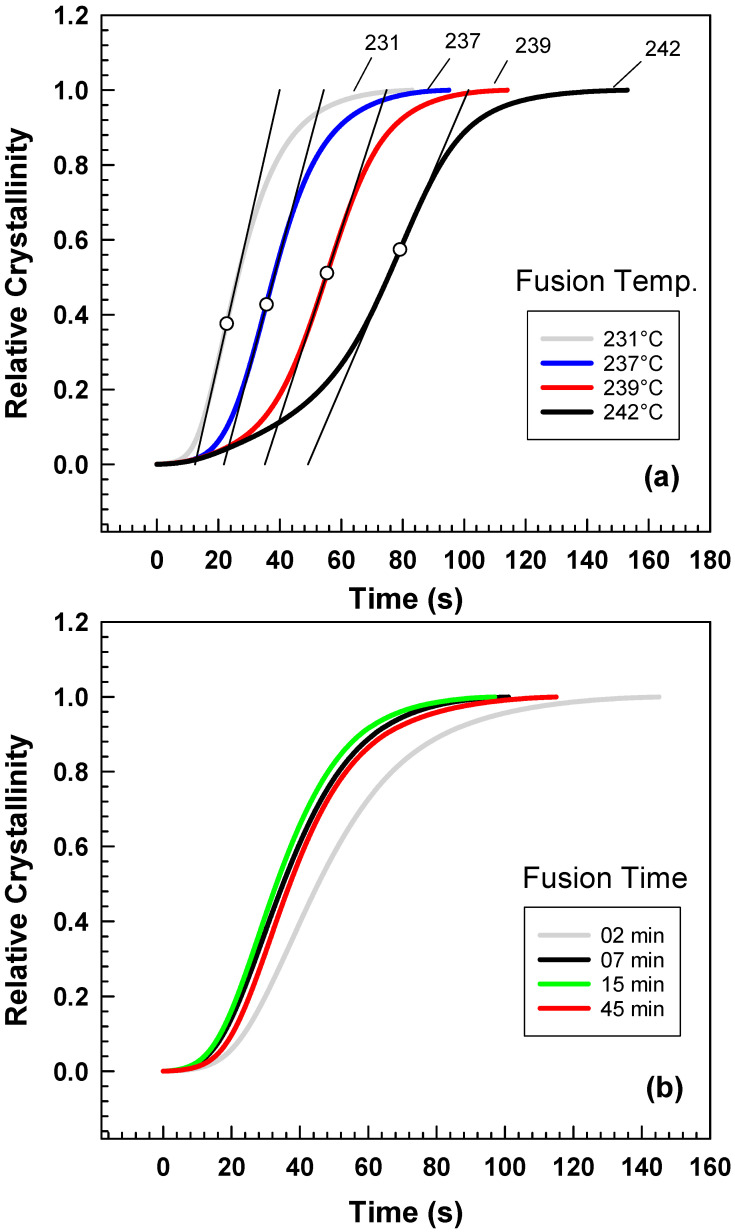
Crystallization kinetics from DSC relative crystallinity vs. time at different (**a**) fusion temperatures (**b**) fusion times.

The experiment assessed the crystallinity of a material through nonisothermal crystallization and computed the corresponding half-life of crystallization. By plotting the relative crystallinity against time for various fusion temperatures, it was observed that there is a positive correlation between the fusion temperature and the crystallization half-life [4,16,32]. Figure 4a depicts a positive relationship between the fusion temperature (range 231–242 °C) for a fixed time of 2 min and the crystallization half-time. The results suggest that an increase in fusion temperature increases the time needed to reach the ultimate crystallinity, i.e., a reduction of the crystallization rate. This can be explained by the thermodynamics of the crystallization process, where higher temperatures promote molecular mobility and crystal growth, leading to a longer time needed to reach a given level of crystallinity. The findings are consistent with previous studies on polymer crystallization kinetics and have significant implications for the processing and properties of polymer materials [3,4,16]. Moreover, the study also investigated the impact of fusion time on the material’s relative crystallinity, as illustrated in Figure 4b.

**Figure 5:**
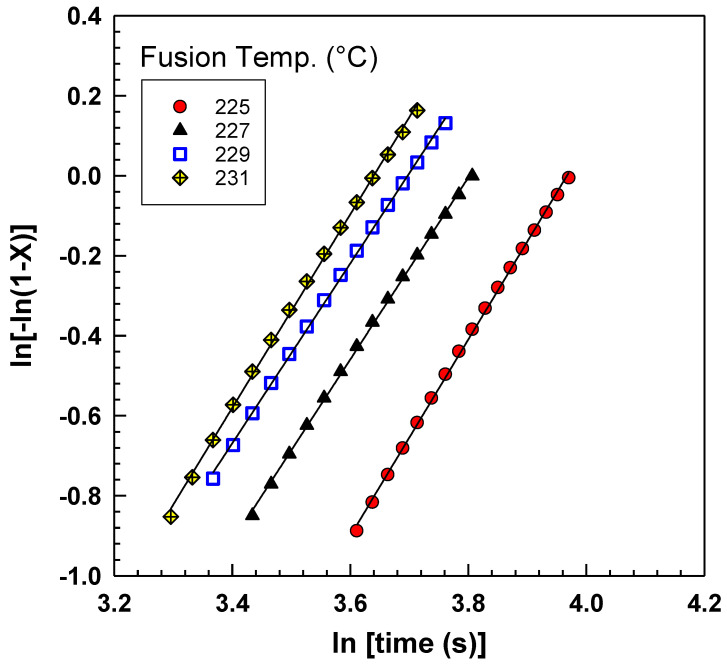
Avrami plot of *ln [−ln(*1 *− X(t))]* versus *ln(t)* at different fusion temperatures.

As described before, the Avrami model [18] is used to study the isothermal crystallization kinetics in polymers. However, using the Avrami model for non-isothermal crystallization has been used by many authors [33,34,35]. Figure 5 shows the Avrami plot of *ln[−ln (*1 *− X(t))]* versus *ln(t)* for PA6 at different fusion temperatures. The Avrami plot shows a linear relationship where R^2^ > 0.999. As can be seen, the Avrami plots exhibit a high degree of parallelism and their temporal positions shift towards lower times as the previous fusion temperature increases [36,37]. The Avrami model has been shown to be highly effective in predicting the nonisothermal crystallization behavior of PA6. This is because the range of temperatures in which crystallization takes place is relatively narrow, meaning that the conditions are not too different from those of an isothermal process. Notably, decreasing a polymer’s fusion temperature can lead to a greater degree of nucleation and faster growth of the crystalline phase [38]. This effect can significantly impact the values of the Avrami parameters, namely *n* and k. Table 2 represents the Avrami parameters *n* and *k* used to characterize the crystallization kinetics. It is evident from the table that both the exponent *n* and the parameter *k* exhibit an upward trend with an increase in the fusion temperature up to 229 °C, following which they display a decline, i.e., limitations of the Avrami model by the fusion temperature of 231 °C because of the presence of shoulder on the heat flow curves after exposure to higher fusion temperatures.

**Figure 6:**
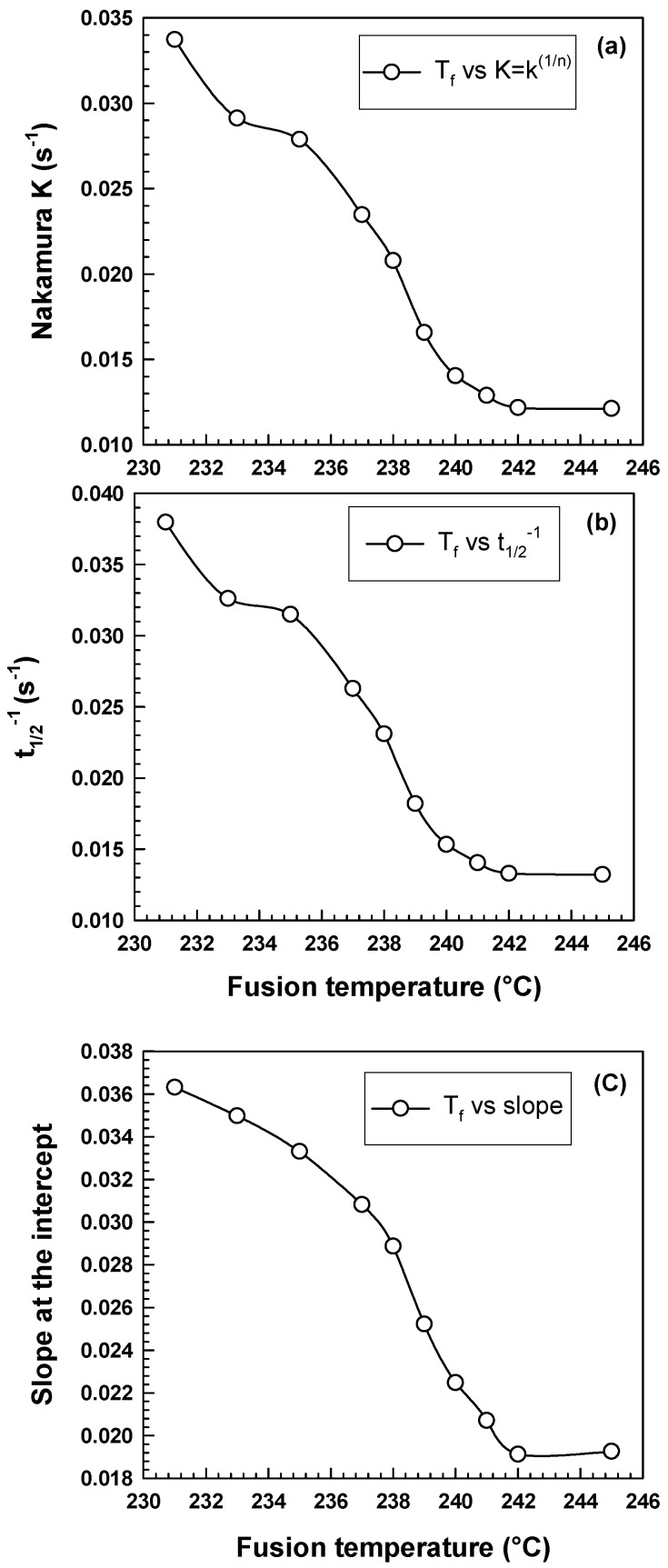
Influence of fusion temperature on the kinetics parameters (**a**) Nakamura *K*, (**b**) *t*_1/2_^−1^, (**c**) slope at the intercept.

However, the Nakamura model [20], proposed in 1973, is a useful way of describing how polymers crystallize when heated. The rate of crystallization is affected by changes in temperature, and a term in the model considers this called the nonisothermal effect. This model has successfully predicted the behavior of many different polymers and has been adapted to account for other factors affecting crystallization. Table 3 displays the Nakamura *K* parameter, demonstrating an inverse fusion temperature correlation. As the fusion temperature increases, the *K* parameter decreases. Higher values of *K* indicate a greater nucleation rate and faster crystallization kinetics [39,40]. This trend is commonly observed in the study of polymer crystallization and has important implications for the processing and properties of polymer materials. The crystallization kinetics of PA6 is highly influenced by changing the fusion temperature [4,37]. Figure 6 and Table 3 show the effect of fusion temperature on (a) Nakamura *K* parameter, (b) inversed crystallization half-time *[t*_1/2_^−1^*]*, and (c) slope at the inflection point. These kinetics parameters are dramatically decreased by increasing the fusion temperature to 242 °C, while higher fusion temperatures (T_f_ > 242 °C) do not affect the crystallization kinetics of PA6.

**Figure 7:**
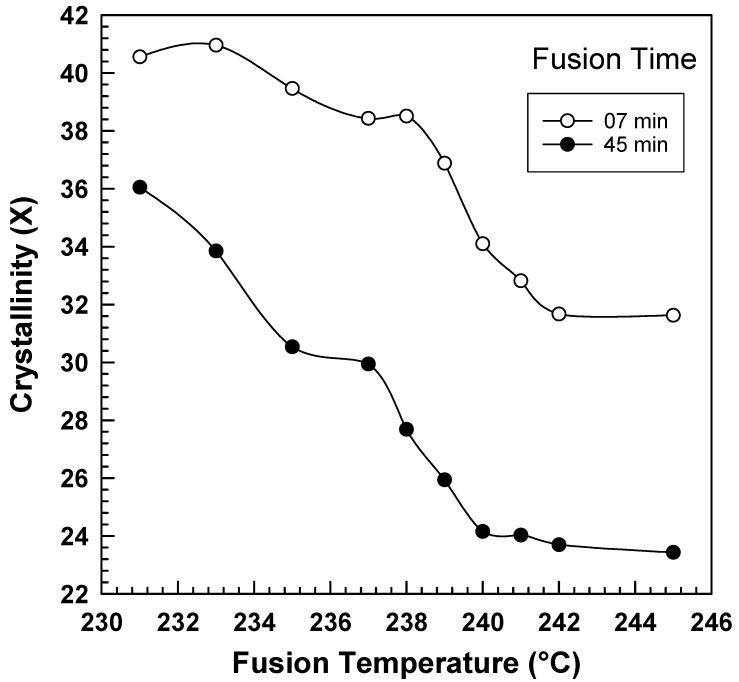
PA6 crystallinity vs. fusion temperature at different fusion times.

The influence of the fusion temperature on the overall crystallinity of PA6 at various fusion times is depicted in Figure 7. It is worth noting that this follows the same pattern as the other kinetics parameters that have been previously discussed. The impact of the fusion temperature on the PA6 crystallization behavior is evident. The information used to construct the figure is also presented in Table 4.

**Figure 8:**
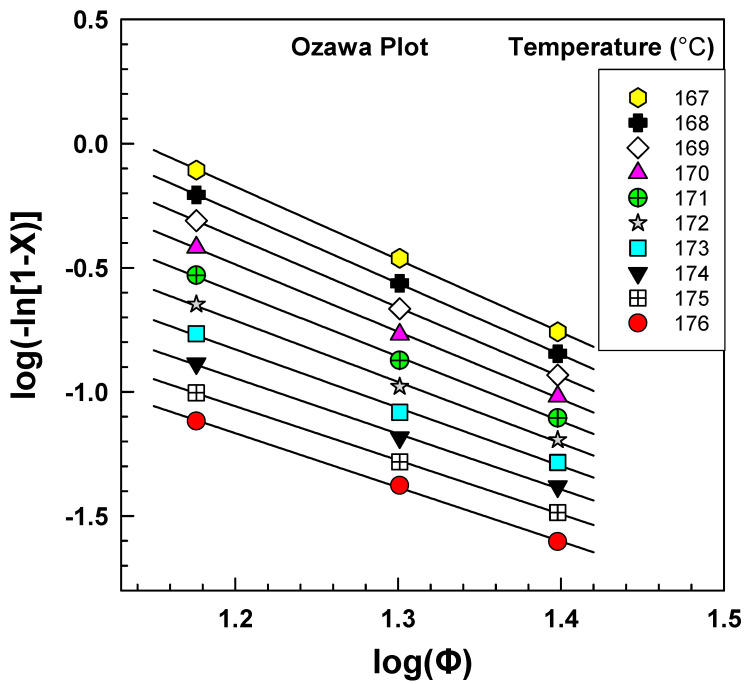
DSC results from the Ozawa plot for PA6.

The crystallinity of PA6 samples was obtained from DSC at a fixed fusion temperature of 239 °C and different cooling rates of 15, 20, and 25 °C/min. Figure 8 shows a linear relationship between *log(−ln [*1 *− X])* and log *Φ*, and the Ozawa model seems appropriate to describe the nonisothermal crystallization of PA6 [19,41]. This linear relationship was obtained due to the narrow range of cooling rates that were used in the experiment. However, other works that used a wider range did not obtain this linearity [16,42]. After constructing the graph (Figure 5) of the dependence of log *[−ln(*1 − *X)]* on log *Φ*, it was possible to obtain the parameters *K* and *m* from the directive of the line or section by linear regression.

Table 5 presents the results of an analysis of the cooling function *K(T)* and Ozawa exponent (*m*) based on the data obtained from Figure 6. The logarithmic cooling function log*K(T)* and the Ozawa exponent were determined from the slope and intercept of the graph and were found to range from 1.449 to 3.349 and 2.2 to 2.9, respectively, with R^2^ values higher than 0.99. The Ozawa cooling functions were calculated at various fusion temperatures ranging from 235 °C to 245 °C. These findings are important for characterizing the nonisothermal crystallization kinetics of the material and provide valuable insight into the thermal behavior of the polymer. Such analyses are commonly used in materials science research to better understand the processing and properties of polymers, and the results can inform the development of new materials with tailored properties.

**Figure 9:**
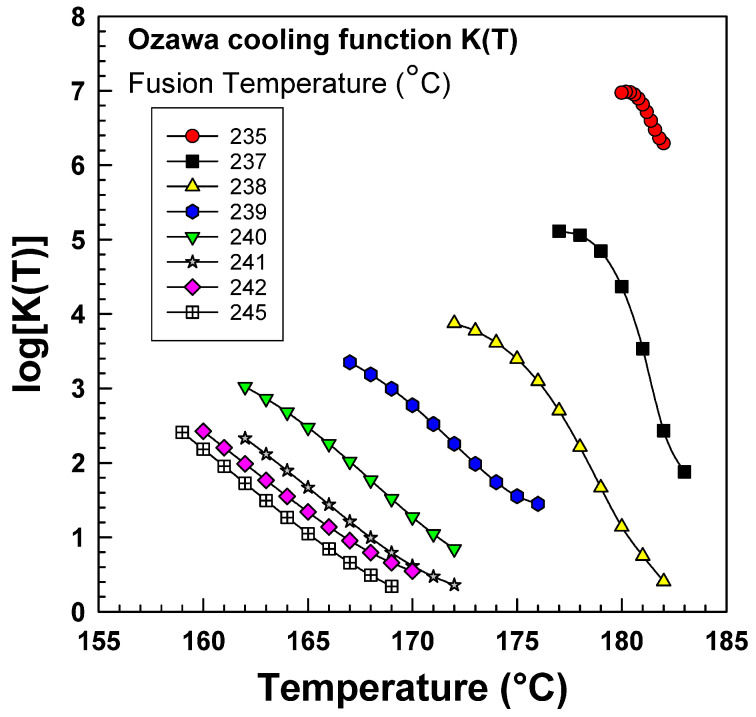
Ozawa cooling function.

Figure 9 illustrates the relationship between the fusion temperature and the Ozawa cooling function *K(T)*. The graph demonstrates that the cooling function *K(T)* decreases and shifts towards the left side as the fusion temperature increases. It is worth noting that the cooling function *K(T)* is a function of both nucleation and growth rate [19]. The graph in Figure 6 illustrates that, for the PA6 polymer, the cooling function *K(T)* increases exponentially as the temperature decreases. This is because as the crystallization temperature decreases, the thermodynamic driving force for crystallization becomes stronger. However, when the temperature becomes low enough, the viscosity of the polymer significantly increases, making it difficult for polymer chains to reach the growth point. This observation is a natural consequence of the PA6 polymer.

Similar *K(T)* dependencies to the crystallization temperature were detected for different fusion temperatures (235 °C to 245 °C). In comparison, the *K(T)* increases while decreasing the fusion temperature. The Ozawa cooling functions of PA6 might result from the number of spherulites generated at different fusion temperatures. A polarized optical microscope analyzed PA6 samples; the samples were heated at different fusion temperatures (235–246 °C) for 2 min and then were cooled down at a cooling rate of 20 °C/min.

As shown in Figure 8, the average number of spherulites decreases with increasing temperature. Raising the temperature from 235 °C to 239 °C reduces the number of spherulites by half. This reduction in the number of spherulites occurs due to the destructive effect of high temperature that reduces the nucleation cores’ production [4].

**Figure 10:**
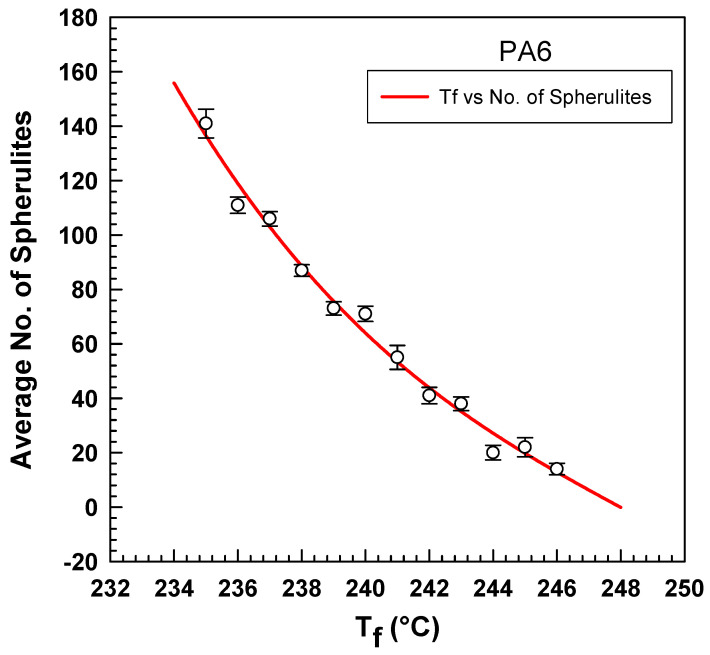
Effect of the fusion temperature on the number of spherulites of PA6 crystallization.

**Figure 11:**
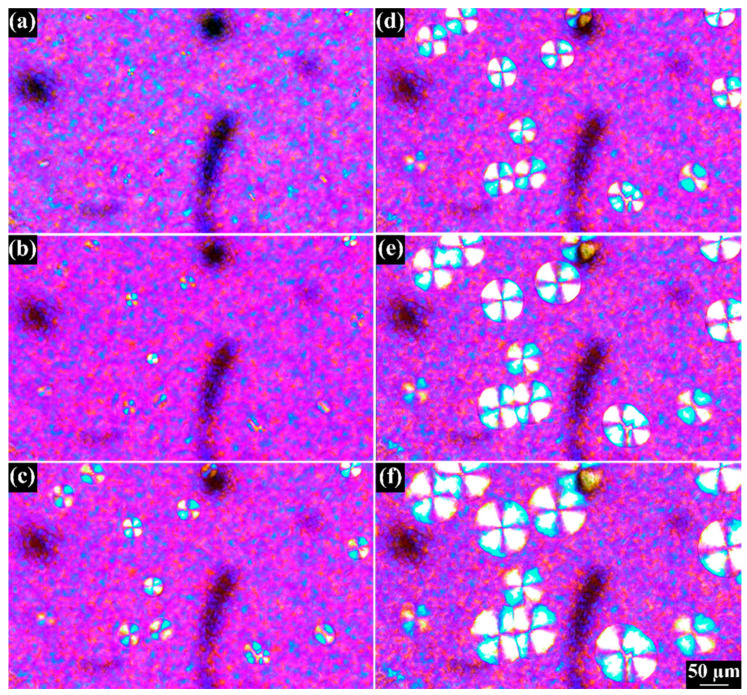
Nonisothermal crystallization by polarized optical microscope at 20 °C/min cooling rate at different temperatures (**a**) 186.3 °C, (**b**) 183 °C, (**c**) 179.6 °C, (**d**) 176.3 °C, (**e**) 173.8 °C, (**f**) 169.8 °C.

Polarized optical microscopy (OM) is used to observe the morphology of PA6 spherulites formed during nonisothermal crystallization. These spherulites are spherical and exhibit highly ordered Maltese cross pattern structures [15,43]. Understanding the details of the spherulite morphology and growth rate is crucial for controlling the final product’s physical properties. Figure 10 presents OM images of PA6 that have undergone a cooling process from 200 °C to 100 °C at a rate of 20 °C/min. During this cooling process, the sample begins to crystallize. The results indicate that spherulites are present during the cooling process. These spherulites have a circular cross-section and exhibit a Maltese cross-pattern system, which suggests that they are oriented along or perpendicular to the crystalline molecular axis concerning the spherulitic radius [16]. The size of the crystallites is highly dependent on the crystallization temperature and time. Figure 11 illustrates the impact of temperature on the rate of spherulite formation during the crystallization process. These spherulite structures are formed due to the presence of many nucleation sites and the rapid cooling of the molten polymer, which impedes normal crystal growth.

**Figure 12:**
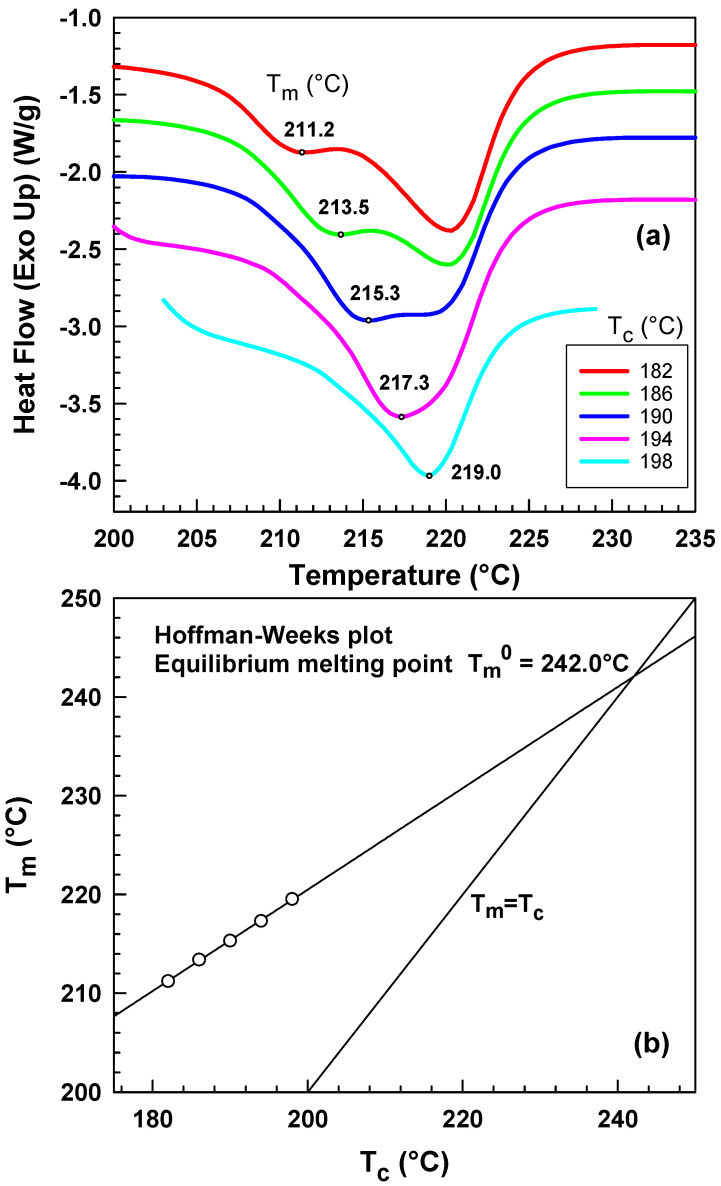
(**a**) Determination of melting temperature after isothermal crystallization at various temperatures, (**b**) Hoffman–Weeks plot for equilibrium melting point determination, melting point of peak I vs. crystallization temperature.

Figure 12 shows the analysis of melting temperature as a function of crystallization temperature that was performed according to the Hoffman–Weeks theory. Where the extrapolated line crosses the *T_m_* = *T_c_* line, there one can find an equilibrium melting point $T_{m}^{0}$. Our $T_{m}^{0}$ was found to be at 242 °C that is very close to Wang et al. who reported equilibrium melting point for pure PA6 to be about 243 °C [44].

## 5. Conclusions

In conclusion, the manuscript studied the influence of fusion temperature and duration on the nonisothermal crystallization kinetics of polyamide 6 (PA6) using differential scanning calorimetry (DSC) and polarized optical microscope (OM). It was found that increasing the fusion temperature led to narrower and lower exothermic traces, resulting in smaller nucleation centers, a shift in crystallization temperature, and a decrease in crystallization kinetics. Additionally, a correlation between the fusion temperature and the crystallization half-time was also observed, indicating that higher temperatures result in longer times needed to reach a given level of crystallinity due to increased molecular mobility and crystal growth. The Ziabicki, Ozawa, and Nakamura models were used to study the crystallization kinetics and found that changing the fusion temperature greatly affected the degree of nucleation and growth of the crystalline phase. The study has implications for the processing and properties of polymer materials.

## Figures and Tables

**Table 1:** Properties of pure materials.

Property	Polyamide 6 F232-D
Melt temperature (10 °C/min)	220 °C (ISO 11357)
Tensile modulus	3300 MPa (ISO 527)
Charpy notched impact strength	6 kJ/m^2^ at 23 °C (ISO 179)
Density	1130 kg/m^3^ (ISO 1183)
Viscosity	214 cm^3^/g (ISO 307)

**Table 2:** Avrami parameters of nonisothermal crystallization of PA6 at different fusion temperatures.

Fusion Temp. (°C)	Avrami Parameters			
	R^2^	Slope	*n*	*k* (s^−1^)
225	0.9992	0.02014	2.45	0.59 × 10^−4^
227	0.9993	0.02313	2.27	1.76 × 10^−4^
229	0.9993	0.02714	2.28	2.29 × 10^−4^
231	0.9990	0.02912	2.48	1.22 × 10^−4^

**Table 3:** Kinetics parameters at different fusion temperatures.

**Fusion Temp. (°C)**	**Nakamura *K* = *k*^(1/*n*)^ (s^−1^)**	***t*** **_1/2_** **^−1^** **(s^−1^)**	**Slope**
231	0.0337	0.0380	0.0363
233	0.0291	0.0326	0.0350
235	0.0279	0.0315	0.0333
237	0.0235	0.0263	0.0308
238	0.0208	0.0231	0.0289
239	0.0166	0.0182	0.0252
240	0.0140	0.0153	0.0225
241	0.0129	0.0140	0.0207
242	0.0122	0.0133	0.0191
245	0.0121	0.0132	0.0193

**Table 4:** Crystallinity of PA6 at different fusion times.

Fusion Temperature (°C)	Crystallinity	
	7 min	45 min
231	40.56	36.05
233	40.96	33.85
235	39.46	30.53
237	38.43	29.94
238	38.51	27.68
239	36.88	25.94
240	34.1	24.15
241	32.82	24.03
242	31.67	23.70
245	31.63	23.43

**Table 5:** Ozawa exponents and cooling functions of PA6.

Temperature (°C)	Slope	Intercept	RSQ
	m	log[*K(T)*]	R^2^
176	2.2	1.449	0.9988
175	2.2	1.552	0.9997
174	2.2	1.741	0.9979
173	2.3	1.986	0.9970
172	2.5	2.255	0.9974
171	2.6	2.521	0.9985
170	2.7	2.770	0.9994
169	2.8	2.993	0.9999
168	2.9	3.186	0.9999
167	2.9	3.349	0.9995

## Data Availability

The data presented in this study are available on request from the corresponding author.
